# “The climate itself must have hidden some medicines”: traditional veterinary medicine of indigenous and non-indigenous campesinos of the southern Andes

**DOI:** 10.1186/s13002-022-00534-8

**Published:** 2022-05-03

**Authors:** Fernanda Olivares, Carla Marchant, José Tomás Ibarra

**Affiliations:** 1 Graduate Program in Rural Development, Universidad Austral de Chile, Valdivia, Los Ríos Region Chile; 2 Laboratory of Territorial Studies LabT UACh, Institute of Environmental and Evolutionary Sciences, Universidad Austral de Chile, Valdivia, Los Ríos Region Chile; 3 ECOS (Ecosystem-Complexity-Society) Co-Laboratory, Center for Local Development, (CEDEL) & Center for Intercultural and Indigenous Research (CIIR), Villarrica Campus, Pontificia Universidad Católica de Chile, Villarrica, Araucanía Region Chile; 4 Department of Ecosystems and Environment, Faculty of Agriculture and Forest Sciences & Center of Applied Ecology and Sustainability (CAPES), Pontificia Universidad Católica de Chile, Santiago, Chile; 5 Cape Horn International Center for Global Change Studies and Biocultural Conservation (CHIC), Puerto Williams, Chile

**Keywords:** Ethnoveterinary medicine, Local ecological knowledge, Medicinal plants, Animal production

## Abstract

**Background:**

Traditional veterinary medicine (TVM) or ethnoveterinary medicine comprises knowledge, practices, and beliefs about farm animals. Its study serves to offer ecologically and culturally appropriate strategies for the management of animals and their health in a context marked by the increased use of synthetic pharmaceuticals, social–environmental degradation, pollution, and climate change. In this study, we examine the TVM that Mapuche and non-Mapuche *campesinos* in the southern Andes have about the management of animals and their health. In addition, we investigate the main factors influencing the current use of TVM.

**Methods:**

Between December 2020 and March 2021, we undertook participant observation and conducted 60 semi-structured and informal interviews with Mapuche and non-Mapuche *campesinos* from the Pucón and Curarrehue municipal districts in the southern Andes of Chile.

**Results:**

We identified a set of knowledge about cycles and manifestations of nature used in planning 14 animal management practices related to a Mapuche *kosmos* expressed in living with respect for and in dialogue with non-human elements. On health management, we recorded knowledge about 30 plant species, whose use for different categories of wounds and parasites has the highest informant consensus factors. The use of these plant species is governed by a *kosmos* associated with respect and reciprocity in their gathering. Nonetheless, 70% of the *campesinos* interviewed prefer to use synthetic pharmaceuticals. We found that the growing use of synthetic pharmaceuticals, the processes of reduction and change in the structure of land ownership, and climate change are perceived as the main factors behind processes of assimilation of new *praxis* and hybridization as well as the reduction and/or loss of the use of TVM.

**Conclusion:**

Our results reveal the presence of ethnoveterinary knowledge, practices, and beliefs that are safeguarded by Mapuche and non-Mapuche *campesinos* in the southern Andes. However, in the context of different social–environmental changes, it is imperative to document, visibilize, and revitalize TVM since it provides new perspectives for bioculturally diverse and sustainable animal production.

## Introduction

Traditional veterinary medicine (TVM) or ethnoveterinary medicine comprises the different knowledge, practices, and beliefs that human groups have about raising farm animals [1]. Ethnoveterinary studies start from the assumption that, over time and through observation, trial, and error, indigenous and non-indigenous *campesinos* have developed their own concepts and techniques for managing animals and their health [1, 2]. TVM is important in human communities whose economic livelihood and food depend on raising domestic animals because it offers viable alternatives for the animals’ care and health, using local resources and without additional monetary cost. Moreover, it has a cultural and symbolic component that is part of the identity of the different peoples [3–6].

Research on TVM is geared to the development of ecological and socioculturally appropriate strategies that consider animal and human well-being [1, 3, 7]. Most research focuses on ethnoveterinary knowledge and practices about using plant species to prevent or cure diseases in domestic animals. In Pakistan, for example, 474 plant species with therapeutic potential in animals have been identified [6]. Similarly, the use of 139 plant species to treat animal diseases has been reported in South Africa [8]. In South America, *campesinos* in Argentina’s semiarid Chaco region use 61 plant species for a total of 81 veterinary treatments [9]. In addition to these knowledge and practices, there are also systems of beliefs that need to be investigated to achieve a comprehensive understanding of animal production, considering aspects of animal management together with its sociocultural particularities [1]. For example, among *campesinos* in Chubut, Argentina, ethnoveterinary practices have symbolic and religious components inherited from Mapuche–Tehuelche ancestors and European Hippocratic medicine [10]. In Los Altos de Chiapas in Mexico, sheep are sacred under the belief system of Tzotzil indigenous *campesino* women and their sacrifice and consumption are forbidden, giving their breeding a meaning beyond its production aspects [11].

Various global phenomena such as changes in ways of life, the intensification of climate change, social–environmental degradation, pollution, the expansion of agrarian modernization, and the adoption of modern veterinary medicine pose a threat to the current and future use of TVM [3, 5, 12, 13]. For example, in livestock farming communities in Zimbabwe, ethnoveterinary knowledge about eliminating external parasites in cattle is considered only in the absence of synthetic pharmaceuticals [14]. Similarly, in Los Altos de Chiapas, Mexico, the use of ethnoveterinary knowledge and practices is threatened due to the impact of population growth and increasing soil erosion on the availability of therapeutically effective plant species [11]. In Catamarca, Argentina, the increasing and priority use of synthetic pharmaceuticals has been cited as posing a risk of the disappearance of ethnoveterinary knowledge and practices [15].

The southern Andes have historically been inhabited by the Mapuche people, who were sovereign and independent from the Chilean nation state until the end of the nineteenth century [16, 17]. In this territory, the human–animal bond dates back to pre-Hispanic times, starting with the hunting of wild birds and the south Andean deer (*Hippocamelus bisulcus*) and the rearing of *chilihueques* (*Lama guanicoe*) and chickens (*Gallus gallus*) before the Mapuche adopted and began to breed the cattle, sheep, horses, and pigs introduced by the Spanish in the sixteenth century [16, 18–20]. The fertile proliferation of the introduced livestock led to the disappearance of *chilihueques* in the seventeenth century and, in the eighteenth and nineteenth centuries, livestock farming became the mainstay of the Mapuche economy [16–18]. In parallel, different accounts from before the nineteenth century report the use of species of trees, shrubs, and herbs for treating human and animal diseases [19–21]. The military occupation of the Mapuche territory by the Chilean nation State at the end of the nineteenth century brought with it a drastic process of dispossession of land, leading to the reduction and deterritorialization of the Mapuche [17, 22]. This, in turn, meant the end of extensive livestock farming and the adaptation of agrosilvopastoral activities to smaller spaces [16, 22]. At the same time, the territory was colonized by migrants from the rest of Chile and overseas who, once settled, used the land for other agrosilvopastoral activities, including particularly wheat farming and forestry [22, 23]. In the late twentieth century, in a territory by then shared by Chileans, foreigners and the Mapuche, the Chilean state promoted a strategy of productive and economic modernization for the Mapuche and non-Mapuche *campesino* sector [24, 25]. This involved the promotion and/or transfer of agricultural and livestock technical advice through extensionist government programs. They included the introduction in 1996 of the Local Action Development Program and, in 2009, the Indigenous Territorial Development Program, both of which remain in operation [24, 26].

Despite the different historical and contemporary processes of profound change that have occurred in the southern Chilean Andes, this territory has acted as a refuge for knowledge about agrosilvopastoral practices, safeguarded by both Mapuche and non-Mapuche *campesinos* [27, 28]. Specifically, in the case of studies of the traditional veterinary medicine practiced there, some research has addressed it from another standpoint. For example, there are studies that indicate the existence of constant dialogue between agricultural activities and the raising of domestic animals that favors ecological interaction, based on the use of animal manure to fertilize home gardens and grassland, as well as the use of animals in cropland to control pests [28–30]. In the case of medicinal plant species, different studies have looked at their use in humans [31–34]. Moreover, studies of TVM have concluded that the repertoire of plant species for human use may also be used in animals [12, 35]. Together with historical records about the use of plants and animal management practices [19, 20] and research that indicates the presence of TVM among *campesinos* of Mapuche–Tehuelche descent in Argentine Patagonia, this suggest the current presence of potential ethnoveterinary knowledge, practices, and beliefs.

This study seeks to answer the following questions: What ethnoveterinary medicine (*corpus* or knowledge, *praxis* or practices, and *kosmos* or beliefs) about the management of animals and their health are found among Mapuche and non-Mapuche *campesinos* in the southern Andes? What are the main factors influencing their current use? To this end, we examine the use of ethnoveterinary medicine for (1) animal management, understood as the different activities involved in caring for farm animals, and (2) ethnoveterinary medicine for health management, understood as the different plant species known for the prevention and treatment of diseases in these animals. Finally, we identify the main perceived factors that influence current use of traditional veterinary medicine by Mapuche and non-Mapuche *campesinos* in the southern Chilean Andes.

## Methodology

### Area studied and ethnographic context

**Fig. 1:**
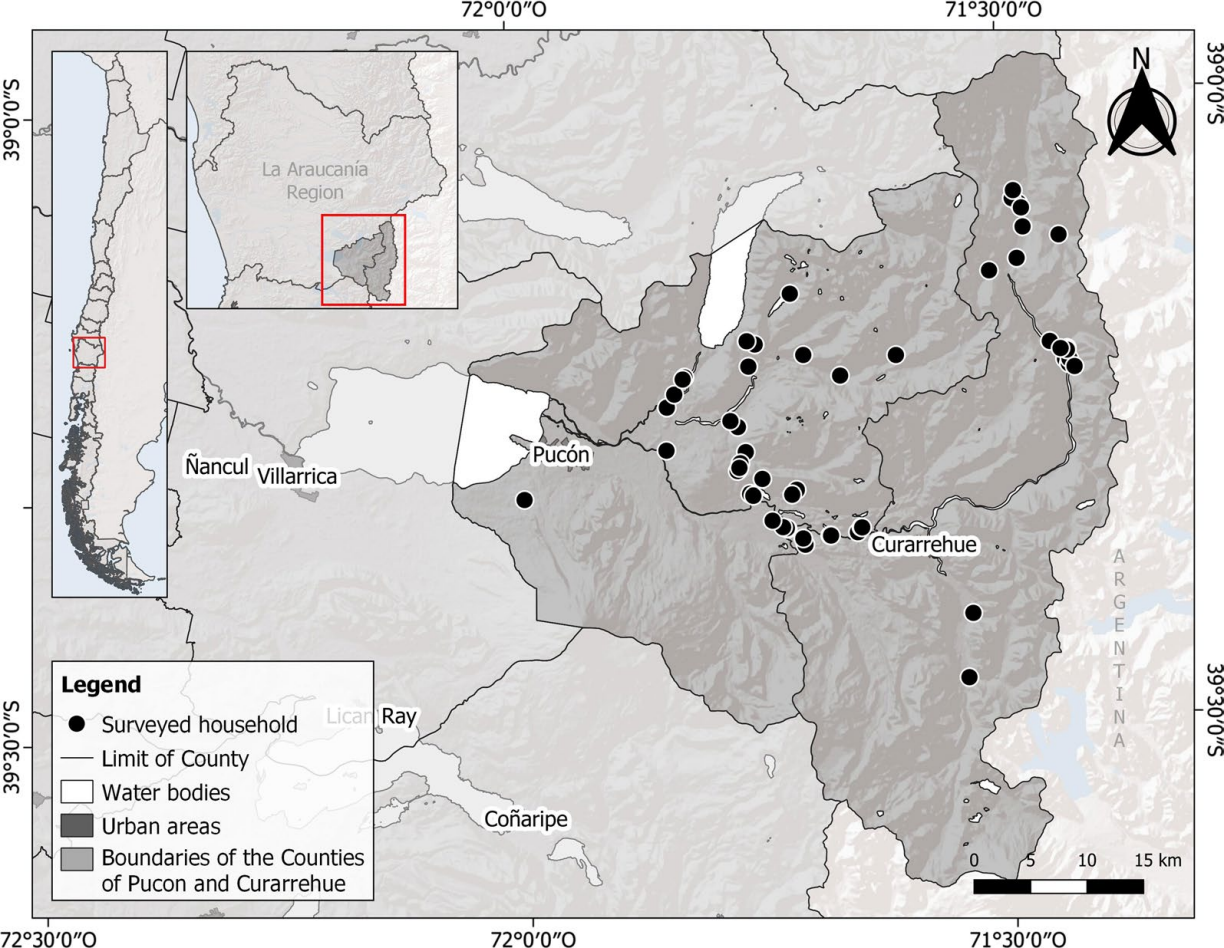
Study area in the Curarrehue and Pucón municipal districts, Araucanía Region, southern Chilean Andes

The study was carried out in the Pucón and Curarrehue municipal districts, located in the Andean part of the Araucanía Region (39° S and 71° W) in the southern Chilean Andes (Fig. 1). The area has a temperate climate, with an average annual rainfall of 2,556 mm. January and February (summer) are the warmest months, with an average temperature of 15 °C in Curarrehue and 16 °C in Pucón. July and August (winter) are the coldest months when the temperature averages 4.9 °C in Pucón and 1.7 °C in Curarrehue. The Pucón municipal district has 28,523 inhabitants of whom 36% live in rural areas and 29% are Mapuche [36], while the Curarrehue district has 7,489 inhabitants of whom 70% live in rural areas and 67% are Mapuche [36]. The territory has examples of large, medium, and small-scale livestock production, including cattle (*Bos taurus*), sheep (*Ovis aries*), goats (*Capra hircus*), pigs (*Sus scrofa*), horses (*Equus caballus*), and/or poultry (*Gallus, Meleagris gallopavo*, and *Anas platyrhynchos domesticus*) [36]. In the small farms of Mapuche and non-Mapuche *campesinos*, different traditional agrosilvopastoral practices are used to produce for family consumption and/or sale as well as for cultural and symbolic purposes [27, 28, 37]. *Mapuzungun* (mapu = land; zungun = speech) is the Mapuche language, spoken by some elderly people and adults but, for most of the territory’s inhabitants, Spanish is their first language and they know only a few words of *Mapuzungun* [37].

### Methodological design and fieldwork

The research used a mixed approach based on quantitative and qualitative methodologies [38]. The fieldwork, which included participant observation and semi-structured and informal interviews, took place in January–March 2021. Interviewees were selected using the snowball sampling method [39, 40]. In all, there were 60 semi-structured interviews with Mapuche and non-Mapuche *campesinos* from Pucón (*n* = 30) and Curarrehue (*n* = 30), conducted after the signing of a letter of free and informed consent, as well as 20 informal interviews. Mapuche and non-Mapuche *campesinos* were grouped together since the latter share the bond of having been born in the territory and of living and working there. Moreover, their agricultural systems incorporate and resemble the traditional Mapuche system [27, 41]. In the first part of the interview, information was obtained about interviewees’ socio-productive characteristics before broaching the knowledge that structures livestock farming (*corpus*) and ethnoveterinary animal management practices (*praxis*) in the different types of livestock farming. The names of the plant species and parts used to treat diseases in animals (*corpus*) were then documented, together with the methods for preparing and administering the treatments (*praxis*). Information was also collected about the system of beliefs (*kosmos*) implicit in both animal and health management. Finally, interviewees were asked about the main factors influencing current animal and health management practices. The semi-structured interviews were complemented with informal interviews and participant observation [39], which were recorded in field notes.

### Data analysis

The interviews were transcribed and subjected to thematic content analysis [42] using the Atlas.ti software. Specifically in the case of animal management, the *corpus* was also classified into categories of knowledge [43]: astronomical, related to the phases of the moon; geophysical, related to the lithosphere (soil types), the hydrosphere (water cycles), and the atmosphere (winds, clouds, climates); biological, related to the use of plants, animals, fungi, and microorganisms; and eco-geographic, related to vegetation and landscapes. The closed interview questions were coded in an Excel spreadsheet for descriptive analysis of frequency, mode and mean with the SPSS program version 23. Additionally, in the case of health management, the informant consensus factor (ICF) was calculated [44] for the different disease categories identified:

$${\text{ICF}} = \left( {{\text{nur}} - {\text{nt}}} \right)/\left( {{\text{nur}} - 1} \right)$$

where nur = number of reports of use in each category; nt = number of species used by category.

The product of this factor fluctuates between 0 and 1, with results close to 1 indicating that the plant species of the group are used by a large proportion of people, suggesting that they are of greater cultural interest. Finally, the results obtained from the quantitative and qualitative analysis of the semi-structured interviews, informal interviews, and participant observation were integrated in order to answer the research questions [38].

## Results

### Socio-productive characterization of Mapuche and non-Mapuche *campesinos*

**Fig. 2:**
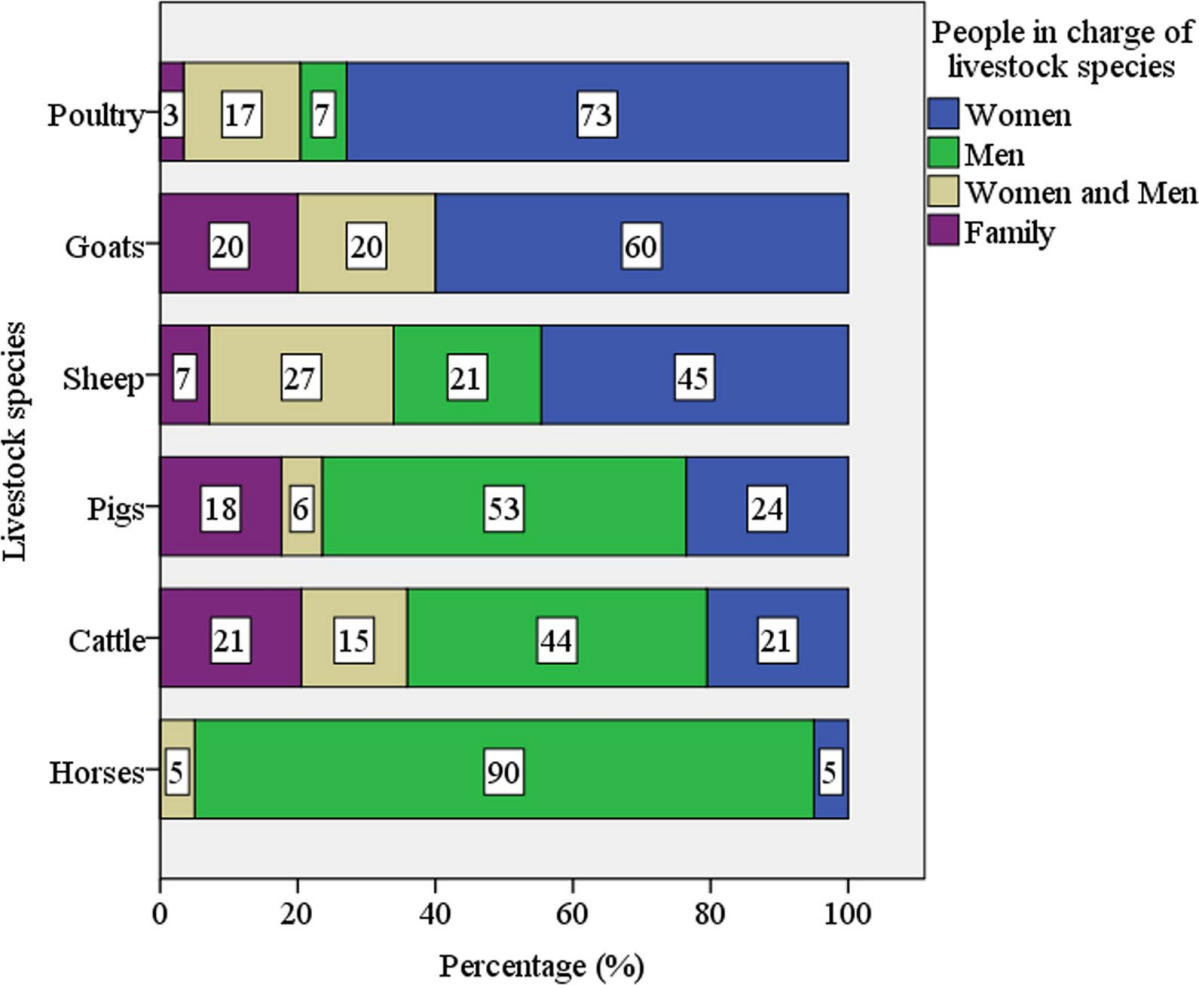
People in charge of different livestock species in the southern Chilean Andes

A total of 60 campesinos (48 women and 12 men) were interviewed of whom 77% were Mapuche and 23% were non-Mapuche. Out of the interviewees, 58% were seniors (≥ 60 years), 40% were adults (30–59 years), and 2% were young people (15–29 years). Three-quarters (75%) were receiving advice through government programs. The area used by interviewees for their different agrosilvopastoral tasks averaged 9.8 hectares (mode of 2 ha). The most frequent types of production—that is, the animals farmed by the largest number of *campesinos* interviewed—were poultry (97%), sheep (93%), and cattle (65%) while horses (33%), pigs (28%), and goats (8%) were less common. As regards the members of the family nucleus responsible for each activity, women were mainly in charge of poultry (73%), goats (60%), and sheep (45%), for which spaces around the home are used during the day and, at night, barns, sheds, or coops (Fig. 2). Men were primarily responsible for horses (90%), cattle (44%), and pigs (53%) (Fig. 2). Horses are kept in areas around the home because they serve as a means of transport while cattle are raised in grazing sites in the mountains, on rented grassland, and on summer pastures (movement of the herd to mountain areas during the summer for grazing and mating). Pigs are raised both near the home and further away.

### Ethnoveterinary knowledge and practices for animal management

Our results indicate the existence of a body of knowledge or *corpus* of the following types: atmospheric (temperature, photoperiod, winds, periods of rain); astronomical (lunar phases); biological (plant and animal species); eco-geographic (landscape and vegetation units); and about the hydrosphere (water courses) and the lithosphere (soil characteristics). This *corpus*, grouped into the different seasons of the year, is used to plan 14 practices or *praxis* related to the raising of cattle, sheep, goats, horses, pigs, and poultry (Table 1). The main practices mentioned by interviewees are described below.

**Table 1:** Corpus and praxis of TVM by Mapuche and non-Mapuche campesinos, southern Chilean Andes

*Corpus*		*Praxis*		
Season of year	Description	Category	Animal management	Description
Winter	*Praxis* organized by *corpus* related with lower grassland productivity, snow in mountainous sectors, and availability of shrubs and/or trees	(AT) (LIT) (BIO)	Supplementary feeding	Feeding animals with bales, concentrate, oats, bamboo (*Chusquea* spp.), and *palo trébol* (*Dasyphyllum diacanthoides*) to supplement production deficit of pastures
	*Praxis* organized by *corpus* represented by temperature minimums, snow in mountainous sectors, abundant rainfall, and the presence of predators	(AT) (BIO)	Animal shelter	The practice of providing shelter for animals in pens, sheds, pigsties, coops, and trees
Spring	*Praxis* organized by *corpus* about increase in temperatures, drop in rainfall, increased grassland productivity, and animal reproduction cycles	(AT) (BIO)	Calving	Planning of calving for different species to reduce neonatal and postnatal deaths associated with feed deficits and extreme weather conditions. The organization of calving permits a supply of animal protein for consumption and/or sale at different times of the year
	*Praxis* planned on basis of *corpus* related to increase in temperatures and the cycle of the *trune* (*Acaena ovalifolia*) and *pimpinela* (*Acaena pinnatifida*) species	(AT) (BIO)	Shearing	Planning of shearing before the *trune* (*Acaena ovalifolia*) and *pimpinela* (*Acaena pinnatifida*) species go to seed in order to prevent these seeds from sticking to the wool
	*Praxis* organized by *corpus* related to the waning phase of the moon and cloudy days with low temperatures during the summer	(AS) (AT)	Tail docking	The tail docking of ewes to facilitate the mating and lambing of future breeding ewes is carried out during the waning moon, which is associated with a decrease in body fluids, preventing hemorrhages and facilitating the coagulation of the cut
Summer	*Praxis* organized by *corpus* about increase in temperatures, drop in grassland productivity, the reproductive cycles, and vegetation units in mountains	(AT) (LIT) (HID) (BIO) (EG)	Cattle transhumance	The practice of transhumance in which cattle are moved to mountainous areas from December to March for feeding and mating
	*Corpus* about the effect of salt in preventing mice from entering the shed where fodder is kept. In addition, adding salt makes the feed more palatable in winter	(AT) (BIO)	Fodder storage	The practice of storing unbaled grass with salt in sheds
	*Praxis* organized by c*orpus* related to the availability of seeds for improving pasture	(AT) (BIO)	Seed harvesting	The seeds of cat grass (*Dactylis glomerata*), clover (*Trifolium *spp.), ryegrass (*Lolium* spp.), and meadow soft grass (*Holcus lanatus*) are selected and harvested for grassland improvement. The seeds are harvested from the grassland as well as from the sheds where fodder is stored
	*Praxis* organized by *corpus* about the reproductive cycles of sheep and increase in temperatures	(AT) (BIO)	Ram separation	The ram is separated from the sheep flock in order to send the ewes to early mating, scheduling lambing for a season with greater forage availability and better weather conditions
	The right time to implement this *praxis* is determined by a *corpus* related to the presence of a waning moon and cloudy days with low temperatures in summer	(AS) (AT)	Castration	The practice of removing the testicles of cattle destined to become steers or bullocks and pigs reserved for fattening during a waning moon
Autumn	*Corpus* related to reduction in hours of daylight, the characteristics of the reproduction cycle of sheep, and reduction in temperatures	(AT) (BIO)	Mating	This practice consists of bringing together the previously separated rams with the flock of ewes for mating in April and/or May
Annual	*Corpus* about the presence of different ectoparasites in poultry	(BIO)	Adding ash to chicken coops	The practice of using ash in chicken coops to control ectoparasites such as bed bugs (*Cimex lectularius*) and red mites (*Dermanyssus gallinae*)
	*Corpus* about the presence of slugs *(Deroceras reticulatum)* in the apex of grass	(AT) (BIO)	Late release of sheep	The practice of releasing the flock of sheep confined in barns, sheds, and/or pens after 9 am to prevent the animals from ingesting slugs (*Deroceras reticulatum*), which cause swelling and death of the sheep
	*Corpus* about the positive effect of animal manure on the soil and the productivity of grassland used for animals	(LIT) (BIO)	Fertilization with animal manure	Incorporation of animal manure into the grassland for fertilization. There are different methods such as: spreading and incorporating the fresh manure left by the livestock when grazing; leaving the sheep manure to dry in sheds and then incorporating it into the meadows; preparing a mixture of manure from different animal species for incorporation into the meadows once it has dried; and mixing animal manure with different organic waste

Nomenclature: Knowledge categories: (AS): Astronomical; (AT): Atmospheric; (BIO): Biological; (EG): Eco-geographic; (HD): Hydrosphere; (LIT): Lithosphere

#### Supplementary feeding

Is employed by 95% of the campesinos who breed cattle (frequency = 37), by 98% of those who breed sheep (frequency = 55), and by 100% of those who breed horses (frequency = 20) and goats (frequency = 5)^1^. It consists of feeding cattle, sheep, horses, and goats with bales of fodder, harvested on the same property or purchased from neighbors and/or external suppliers during the summer. Supplementary feeding with grains and/or concentrate is used mainly for horses, pregnant ewes, and thin cattle. These supplements are acquired from external suppliers and/or as benefits for *campesinos* registered with government programs. Interviewees indicated a preference for supplementing with oats, rather than concentrate, given the versatility of oats as feed for different species as well as its effect on the animals, as reported by one interviewee: “*The concentrate makes the sheep’s babies develop a lot and then they have trouble giving birth; the same happens with cows, that’s why oats are better*” (man, 74 years). We also identified supplementary feeding with bamboo (*Chusquea* spp.), branches of the *palo trébol* tree (*Dasyphyllum diacanthoides*), and the addition of salt to cattle and sheep feeders.

#### Sheep mating

**Fig. 3:**
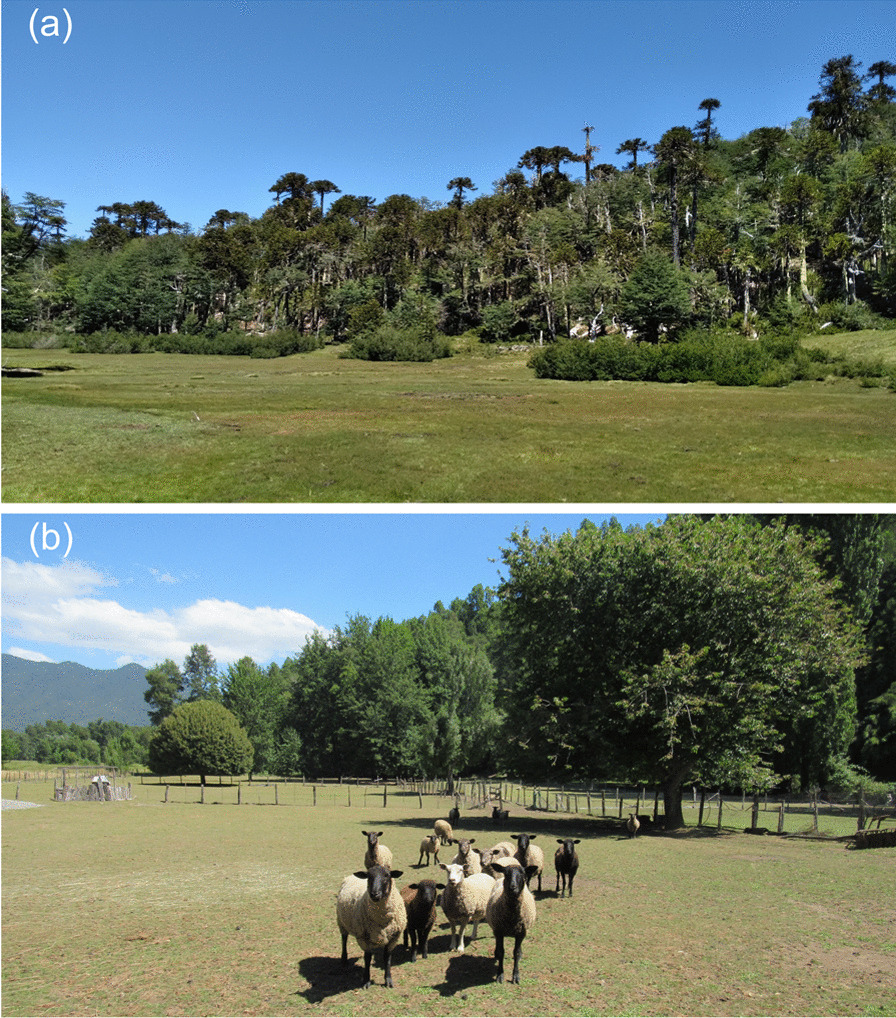
Images showing **a** summer grazing area (“veranada”); **b** sheep flock next to rams

Mating, using a ram previously separated from the flock, takes place in April and/or May, as indicated in the following account: “*I learned from the elders who separated the ram, they mate in early May so birth is in October*” (man, 75 years) (Fig. 3). The separation of the ram is practiced by 48% of the *campesinos* with sheep (frequency = 27), scheduling lambing for a time of year when more forage is available and the weather is better.

#### Putting ash in chicken coops

This practice is used by 46% of *campesino* women who keep poultry (frequency = 27). It consists of putting piles of ash from wood-burning stoves or fireplaces inside the chicken coop, as explained by the following interviewee: “*I put ash in the chicken coops so they roll in it and get rid of bed bugs*” (woman, 68 years). The ash would prevent infestation by hematophagous arthropod-type ectoparasites which cause economic losses due to a reduction in egg laying, weight loss in the chickens, and, in severe cases, even their death.

#### Summer grazing

It is used by 41% of the *campesinos* who farm cattle (frequency = 16). The animals are left on their summer pastures until April–May when they are brought down to winter pastures or places closer to the owner’s home*.* During the summer, the herd is visited intermittently by its owners or those responsible for it, who are mostly men. Summer grazing means that the animals have access to different forage species, unlimited water sources, and trees for shelter, as indicated by one interviewee: “*The animal grows healthier in the mountains than anywhere else… because it drinks good water… when the calves are brought down, they look like fat little barrels, because the cow produces good milk, because she eats good herbs*” (man, 85 years). In their summer grazing, the animals have access to functional forage—or, in other words, food that is perceived as positive for animal health and yield—as well as medicinal forage, that is, food eaten while foraging that prevents and cures diseases [45]. The *campesinos* perceive that the animals prefer to graze in *mallínes,* a type of wetland meadow*,* where they have access to functional types of forage (Fig. 3). The medicinal forage mentioned includes *paramela* (*Adesmia boronioides*) and quinchamali (*Quinchamalium chilense*), herbs with therapeutic properties that are part of the Mapuche medicinal repertoire.

#### Castration

This practice consists in removing the testicles of cattle destined to become steers or oxen and of pigs for fattening. The presence of a waning moon determines the appropriate moment for this *praxis* and is considered by 41% of the *campesinos* with cattle (frequency = 16) and 35% of those with pigs (frequency = 6). The waning moon is associated with a decrease in body fluids, as indicated in the following account: “*When the moon is finishing its waning, the animals are castrated, because they bleed less; when the moon is waxing, they bleed a lot*” (woman, 73 years). In this way, hemorrhages are avoided and clotting and healing are facilitated.

### Ethnoveterinary knowledge and practices for health management

Our results identify a *corpus* represented by 30 plant species from 24 botanical families with therapeutic potential for preventing and treating diseases in cattle, sheep, goats, horses, pigs, and poultry. In most botanical families, only one species was identified, except for *Asteraceae*, *Rosaceae*, *Lauraceae*, *Proteaceae*, *Santalaceae*, and *Cunoniaceae* of which there are two species (Table 2). The species identified are mostly of native origin (21 species; 70%; Table 2). The main group are herbs (15 species; 50%), followed by trees (10 species; 33%) and shrubs (5 species; 17%; Table 2).

**Table 2:** Ethnoveterinary plant species mentioned by Mapuche and non-Mapuche campesinos, southern Chilean Andes

Scientific name^1^	Local name	Mapuche name	Family	Origin^2^	Life form^3^	Use form^4^	Species^5^	Frequency^6^
*Drimys winteri* (J.R. Forst. & G. Forst.)	Canelo	*Foye, Foique*	Winteraceae	N	T	P; I	C, H, P, S	24
*Juncus procerus* (E. Mey)	Hierba de la vaca, juquillo		Juncaceae	N	H	R	C	20
*Plantago lanceolata* L	Siete venas		Plantaginaceae	E	H	I; W	C, G, H, P, Pi, S	10
*Acaena ovalifolia* (Ruiz & Pav.)	Trun, trune		Rosaceae	N	H	D	C, S	8
*Weinmannia trichosperma* (Cav.)	Palo santo	*Teñiu, Maden*	Cunoniaceae	N	T	R; D	C, S	7
*Cryptocarya alba* (Molina) Looser	Peumo	*Pengu*	Lauraceae	N	T	P; D	C, S	6
*Lomatia hirsuta* (Lam.) Diels	Radal	*Radal, Raral*	Proteaceae	N	T	D; R	C, S	6
*Azara serrata* (Ruiz & Pav.)	Corcolén		Flacourtiaceae	N	S	I; P	C, H, S	5
*Buddleja globosa* (Hope)	Matico	*Pañil, Palguin*	Scrophulariaceae	N	S	W	C, G, H, P, Pi, S	5
*Fragaria* × *ananassa*	Frutilla		Rosaceae	N	H	R	C	4
*Aristotelia chilensis* (Molina) Stuntz	Maqui	*Maki, këlon*	Elaeocarpaceae	N	T	D; I; M	C	4
*Chenopodium quinoa* (Willd.)	Quinoa	*Quinwa*	Amaranthaceae	N	H	I	P	4
*Caldcluvia paniculata* (Cav.) D. Don	Triaca	*Quiaca, Mepua*	Cunoniaceae	N	T	I; R	C, S	4
*Alstroemeria aurea* (Graham)	Liuto		Alstroemeriaceae	N	H	M	C	2
*Myoschilos oblongum* (Ruiz & Pav.)	Orocoipo		Santalaceae	N	S	D	C, S	2
*Chenopodium ambrosioides* L	Paico		Chenopodiaceae	N	H	D	S	2
*Artemisia absinthium* L	Ajenjo		Asteraceae	E	H	I	H	1
*Capsicum annuum* L	Ají		Solanaceae	E	H	I	P	1
*Allium sativum L*	Ajo		Liliaceae	E	H	I	P	1
*Nothofagus dombeyii* (Mirb) Oerst	Coihue	*Coihue*	Fagaceae	N	T	D	C	1
*Mentha spicata* L	Hierba buena		Lamiaceae	E	H	R	C	1
*Foeniculum vulgare* (Mill.)	Hinojo		Apiaceae	E	H	D	S	1
*Laurus nobilis* L	Laurel de campo		Lauraceae	E	T	I	P	1
*Helianthus annuus* L	Maravilla		Asteraceae	E	H	P	P	1
*Gunnera tinctoria* (Molina) Mirb	Nalca	*Pangue*	Gunneraceae	N	H	R	C	1
*Embothrium coccineum* (J.R. Forst. & G. Forst.)	Notro		Proteaceae	N	T	E	C, S	1
*Ribes sp.*	Parrilla	*Mulul*	Grossulariaceae	N	S	M	C	1
*Chusquea sp.*	Quila, coyocho	*Cüla, colew*	Poaceae	N	S	E	C, S	1
*Quillaja saponaria* (Molina)	Quillay	*Cüllai, Quillay*	Quillajaceae	N	T	I	S	1
*Quinchamalium chilense* (Molina)	Quinchamalí		Santalaceae	N	H	D	C	1

^1^Plant author names are given only for species identified at the species level. Nomenclature: ^2^Origin: N = Native; E = Exotic. ^3^Life form: A = Tree; S = Shrub; H = Herb. ^4^Use form: D = Diarrhea; W = Wounds; I = Infection; M = Mastitis; P = Parasites; Q = Eye disease; R = Retained afterbirth. ^5^Species: C = Cattle; G = Goats; H = Horses; P = Poultry; Pi = Pigs; S = Sheep.^6^Frequency: Number of mentions of each plant species

We identified seven categories of symptoms and diseases (Table 3). The systemic infections category groups together diseases caused by different etiological agents. Their symptoms are a fever and general unwellness, with the latter also referred to as *achaque* or *tristeza* (sadness) in the animal. The diarrhea category includes conditions commonly known as *empacho* (indigestion) or *churreta* (the runs). Placental retention is the failure to deliver the afterbirth and is also known locally as “*no botar pares*.” The parasites category includes infestation with internal and external parasites while the mastitis category refers to the symptoms associated with udder inflammation in ruminants. Finally, the keratoconjunctivitis category includes diseases that cause blindness or cloudy eye in ruminants. The categories of wounds (ICF = 0.91) and parasites (ICF = 0.90) have the highest values, suggesting common use of the species included in these groups, due probably to their cultural importance and bioactive potential (Table 3).

**Table 3:** Informant consensus factor (ICF) by animal disease category mentioned by Mapuche and non-Mapuche campesinos, southern Andes

Disease/symptoms category	Nº of species used	Nº of reported uses	ICF value
Infectious diseases	11	17	0.38
Diarrhea	10	25	0.63
Retention of placenta	7	36	0.80
Mastitis	3	4	0.33
Wounds	2	12	0.91
Parasites	4	31	0.90
Eye disease	2	2	0.00

The techniques used to prepare the treatments are decoction (42%), crushing (23%), infusion (14%), direct administration (12%), drying leaves (2%), heating stems (2%), soaking seeds (2%), and burning tree bark (2%). For decoction, infusion, and soaking, the solvent used is water to which, depending on the disease in question, ash, soot, sugar, and/or salt may be added. The preparations are administered orally (77%), topically (18%), ophthalmically (3%), or as smoke (3%).

### Ethnoveterinary system of beliefs

We identify a system of beliefs closely linked to the Mapuche *kosmos*, which is expressed in participation in the *Nguillatun* ceremony as well as in the daily way of inhabiting the territory. The *Nguillatun* (Nguillatu = ask; *n* = action) is a supplication held every two to three years by Mapuche communities in which a connection with the *Wenu Mapu* (highlands) is established. In this way, dual energies are balanced through supplication and thanksgiving to *Ngentechen* (God), begging for different living manifestations, as reported by an interviewee: “*We ask for everything, for health, the harvests, for this illness that is around, for the animals*” (woman, 81 years). This reflects an integrative vision that takes into account the welfare of all the beings that inhabit the earth, maintaining balance and, therefore, well-being. Despite the importance attributed to the *Nguillatun*, not all the Mapuche *campesinos* interviewed participated in it, due to factors such as loss of the tradition because of the death of ancestors, distance from the places where the ceremony takes place, and, in some sectors, a larger proportion of young people, who do not identify with it. Non-Mapuche *campesinos* living in neighboring sectors indicated that they were aware of the ceremony but did not take part in it because they did not feel Mapuche. The presence of Catholic and Evangelical churches in the territory indicates the existence of a system of beliefs linked to these religions. Members of Mapuche communities may identify with Catholicism while remaining Mapuche. By contrast, Evangelical churches seek to replace traditional Mapuche beliefs and ceremonies [46].

When referring to their way of life, Mapuche *campesinos* recount how, in their daily praxis, they ask permission from the *ngen* (the caretakers or owners of places) when, for example, they go into a wood or up a mountain, when livestock is going to be moved, or when crossing a stream. This practice was described by an interviewee: “*Because when you enter land that you have never stepped on before, you ask for permission, or when you enter a small stream and go by that stream, you ask for that water and wet your hand because all those things have their ngen, that’s why one has to ask permission*” (woman, 71 years). This corresponds to the *Az Mapu* or the norms that order reciprocity or, in other words, the space where it is possible to achieve exchange to maintain the dual balances existing in the *kosmos* and, therefore, harmony [47]. In this way, the *Az Mapu* establishes a framework of behavior that is inextricably linked to how animals are raised and is the *kosmos* dimension of ethnoveterinary knowledge and practices.

In the case of health management, it is important to bear in mind that, for the Mapuche, the different plant species are manifestations of life that have a *pullu* (soul) and are governed by a set of rules that determine their use because each plant has a higher owner [34]. In this way, the *Az Mapu* remains present in ethnoveterinary knowledge and practices, establishing principles based on asking for permission and reciprocity when using a plant species, as described by an interviewee: “*One asks for permission, one says ‘excuse me’ because I’m going to take this remedy and hopefully it goes well … permission is asked for all the remedies that one looks for in the countryside … you have to have faith that it will do you good; otherwise, it won’t do anything*” (woman, 71 years). Other interviewees also mentioned the importance of having faith in the effectiveness of the plant species and planning for which illness it is to be used. We observed that, for non-Mapuche *campesinos*, the *praxis* of obtaining a plant species, for use either in humans or animals, takes place in a framework of respect and trust. This can be attributed to the deep-rooted traditions around plant species that exist in the territory where practices are shared, due to the proximity of farms, the human relationships that are generated, and the distance from shopping centers and/or medical centers.

Finally, the notion of *kosmos* is behind the Mapuche conception of illness, as expressed by one interviewee: “*That we get sick, that animals get sick, is something natural, it’s something that’s included in our nature*” (woman, 53 years). It is important that illness is seen as part of the inherent duality of being on earth. In this way, both the use of plant species and the *Nguillatun* contribute to living and acting in equilibrium, in a context marked by constant destabilizing influences. However, this does not preclude attributing illness in animals to human behavior in the form of polluting *praxis* such as the accumulation of waste and the use of agrochemicals, a perception shared by both Mapuche and non-Mapuche *campesinos*.

### Factors influencing current use of traditional veterinary medicine

Our results indicate that factors associated with agricultural modernization processes, reductions and changes in the structure of land ownership, and climate change are perceived as forces affecting current use of traditional veterinary medicine (TVM). They imply the assimilation of new practices and hybridization as well as TVM’s interruption and/or loss. In the case of animal management, the assimilation of new modernizing *praxis* is seen principally in the use of supplementary feeding based on the introduction of energy feeds, technological packages of hybrid seeds and chemical and/or agrochemical fertilizers. These products, introduced through extensionist government programs, are intended to establish pastures for animal supplementation and field clearing as recounted by one interviewee: “*INDAP* [an extensionist government program] *brought the chemicals. We were implementing a project and it was going to buy so much seed and so much fertilizer… but that was the evil that was put in the ground, because now it doesn’t produce natural grass, just weeds*” (woman, 55 years). In this way, TVM practices associated with the harvesting of grass seeds and their subsequent sowing integrated with animal manure (Table 1) are displaced by modernizing practices.

In the case of health management, agricultural modernization is reflected in the introduction and assimilation of the synthetic pharmaceuticals used in modern veterinary medicine. Participants perceived that the increasing use of synthetic drugs has caused the loss of ethnoveterinary medicine, as mentioned by one interviewee: “Before, the Mapuche used only herbal remedies for the cows, they used the palo santo bark for diarrhea… now we use only the remedy that you buy at the vet store” (woman, 80 years). In our study, 70% of interviewees were using exclusively synthetic pharmaceuticals in the form of antiparasitics, vaccines, antibiotics, and vitamins; 14% were applying mixed management, combining TVM with synthetic pharmaceuticals; 11% were using only TVM; and 5% were not using any treatment at all.

According to research participants, reductions and changes in the structure of land ownership are decreasing the use of TVM and are reflected in the disappearance of some types of livestock production and a reduction in different species of animals in response to the more limited space now available, as reported by one interviewee: *“Almost no one here has cows now. Because people sold land and it got smaller… if you walk down, you see houses on the different sides… there isn’t space for raising* [animals] *anymore”* (woman, 70 years). In addition, the change in the structure of land ownership restricts rotation and the movement of livestock between the mountains and grasslands traditionally used for grazing. This is also conducive to a reduction in livestock and increasing dependence on external sources of feed in the form of rented pastureland and/or the purchase of fodder.

In health management, changes in the structure of land ownership have impeded access to places where therapeutic plant species grow, as explained by one interviewee: “*Now I don’t go to collect the palo santo I used to collect higher up, without any restrictions … I would go through a couple of fences and that was it … no longer, because there are locked gates, you can’t go into someone else’s place*” (woman, 53 years). Similarly, anthropic intervention of rivers and the urbanization of rural areas have led to the disappearance of plant species used to treat animals. Climate change also causes a reduction in livestock and, therefore, the space devoted to animal management *praxis*. This is related to a drop in the productivity of the fields used to feed animals, as indicated by one interviewee: *“It rains less and that is greatly affecting people’s way of life, of working the fields, sowing, because if there is no irrigation and it doesn’t rain, you can’t sow … the grass doesn’t grow”* (woman, 53 years). The decrease in rainfall and the effect on growth of the grass reduce fodder availability, leading to the assimilation of new animal feeding *praxis* and hybridization and, in extreme cases, a reduction in livestock. Similarly, in the case of health management, climate change is affecting the availability of therapeutic plant species as reported by one interviewee: “*The climate itself must have hidden some medicines. There are many medicines that have disappeared… And these mountain ranges had them*” (woman, 69 years). Interviewees also noted that high temperatures make animals more prone to diseases and external parasitisms due, for example, to an increase in the presence of horn fly (*Haematobia irritans*) in cattle, situations that are dealt with using synthetic pharmaceuticals.

## Discussion

This study examined how the links between *campesinos*, domestic animals, and their environment generate a traditional veterinary medicine (TVM) that is dynamic, in constant adaptation and closely linked to the Mapuche *kosmos*. It permits the development of different types of livestock farming in respectful dialogue with nature and the non-human elements that support the ethnoveterinary *corpus* and *praxis* and give them their identity. Preliminarily, we identify perceived socio-environmental changes that are affecting these links, including agricultural modernization, reductions and changes in the structure of land ownership, and climate change. These changes are perceived by interviewees as forces leading to a reduction and/or loss of TVM as well as the assimilation of new *praxis* and hybridization.

On animal management, we show how a critical winter season in production terms determines supplementary feeding and animal shelter practices. This makes the *campesinos* more receptive to new practices, related principally to the modernization promoted by government programs. These new *praxis* are incorporated through processes of assimilation and hybridization [32, 48]. A similar case is seen in *campesino* communities in Argentine Patagonia where government programs are introducing new horticultural practices, giving rise to processes of innovation, hybridization, and/or the loss of traditional horticultural knowledge [49]. The assimilation of new practices and hybridization do not necessarily preclude the use of TVM; rather, they correspond to a process of constant evolution and adaptation to changing environmental and socioeconomic conditions [50]. Around the world, indigenous peoples and local communities are resorting to a series of responses to the impacts of climate change and, in the case of the livestock sector, one of the most important responses is the adjustment of feeding practices [51].

Modernizing *praxis* are promoted by extensionist government programs that encourage the use of technological packages to maximize agricultural productivity [27, 30]. Nonetheless, our study shows that the assimilation of new *praxis* and/or hybridization are subject to a *campesino corpus*. For example, the persistence of the use of animal manure as a fertilizer corresponds to a *corpus* and *praxis* based on observation, experimentation, and evaluation of how the soil reacts to chemical fertilizers. This also reflects a *kosmos* that views the soil as a living entity. The *corpus, praxis,* and *kosmos*, together, influence the optimal choice for the fertilization of grasslands. There are, for example, differences in how the soil is conceived by the *campesinos* (as a living entity) and by extensionist government programs (in terms of productivity). These differences reflect different realities and ontologies [52].

Out of the different types of livestock farming covered by our study, poultry is the most representative and is, moreover, a primarily women’s space. This can be related to the paramount role that women, as the persons responsible for domestic tasks, care work, and the management of home gardens, play in the family’s daily sustenance [53, 54]. In this context, poultry farming takes place close to the home, without altering the productive axis formed by home–farmyard articulation. In animal management, this articulation is apparent in the use of ash from the kitchen to prevent external parasites in the poultry. This *praxis* is also seen among *campesinos* in Zimbabwe [14]. The greater prevalence of sheep farming over cattle farming appears to be related to the size of the farm which, as a result of processes of reduction and change in the structure of land ownership, restricts the raising of cattle [16, 22]. Cattle farming appears to be linked to the possibility of access to the *praxis* of summer grazing, the mountains, or rented pastureland. In addition, the maintenance of the *praxis* of transhumance is associated with its productive, cultural, and symbolic value in which the Mapuche *kosmos* is expressed and transmitted intergenerationally [55]. Changes in types of livestock production may also be a response to climate change. For example, in Argentine Patagonia, *campesinos* use physical and animal behavior ethnoindicators to predict short and long-term processes of environmental change as a basis for planning and managing their different types of livestock farming [56]. In this sense, a decrease in livestock production and changes in both the species farmed and the location of pastures are part of one of the main responses of indigenous peoples and local communities to climate change [51].

In the case of ethnoveterinary health management, we identify a *corpus* of more than 30 plant species with therapeutic properties. The categories of diseases and symptoms with the highest informant consensus factors (ICF) are wounds and parasites. A high ICF for the parasite category was also recorded on Colares Island, Brazil, where 56 plant species were identified [57]. Similarly, in Ethiopia’s Ankober district, the highest ICFs among the 51 plant species identified were for gastrointestinal diseases and parasites [58]. In the foothills of the Himalayas in Pakistan where 126 plant species were recorded, the highest ICFs were for the categories of respiratory and reproductive illnesses and parasites [12]. High ICFs for common categories and, in this case, parasites reveal how, in different parts of the world, the knowledge and practices of *campesinos* and indigenous peoples are able to treat common ailments with the resources available locally.

In the ethnoveterinary health management *corpus*, the prevalence of native species can be attributed to knowledge accumulated since pre-Hispanic times. Their medicinal and spiritual effects have been subject to different processes of observation and experimentation, with the consequent development of a medicinal repertoire [21, 31–33]. The assimilation of exotic species has served to diversify the local therapeutic heritage [59] and they are widely used by both Mapuche and non-Mapuche *campesinos* [31–33]. These exotic species are also used for ethnoveterinary purposes by *campesinos* and indigenous communities in other parts of the world. For example, garlic (*Allium sativum*) is used by China’s Buyi people to treat infections in goats and sheep [13]; in Canada, in horses with respiratory, infectious, and digestive problems [60]; and, in Austria and by China’s Nu people, in cattle with parasites, reproductive disorders, fever, and diarrhea [5, 45].

This study found that only a small percentage of *campesinos* use TVM for health management. This can be attributed mainly to the adoption of synthetic pharmaceuticals. Among the Maasai people of Africa, for example, the increasing use of synthetic pharmaceuticals has also been identified as a cause of a decline in TVM use [61]. The situation is different in Argentine Patagonia where the influence of synthetic pharmaceuticals is limited by the area’s geographic remoteness and the resulting difficulties in terms of access to urban centers and technical visits [10]. Although an increase in the use of synthetic pharmaceuticals is reported, a *kosmos* related to respect, trust, and reciprocity in the use of plant species persists. In other words, the Mapuche *campesinos* adopt these new approaches without abandoning their identity [52]. In their case, trust in ethnoveterinary treatments seems to have acquired a hybrid nature, influenced by the Mapuche–Tehuelche *kosmos*, modern veterinary medicine and Christian symbolisms [10]. Other peoples also perceive changes in land use and climate change as threats. For example, in the Lesser Himalayas of Pakistan, the expansion of agricultural land is perceived as the main threat to the ongoing existence of plant species used in ethnoveterinary health management [12]. Similarly, in Nigeria, factors related to urbanization and climate change are reducing the availability of plant species for ethnoveterinary purposes to the detriment of their current and/or future use [62].

Different studies have examined the TVM of *campesinos* as offering approaches and alternatives that are resilient, harmonious and respectful of nature [1, 7, 10]. This research has shown how *campesinos* in different parts of the world implement strategies of adaptation to climate change through the incorporation of animal manure [63, 64]. It has also identified characteristics of plant species that serve as ethnoindicators of short and long-term climate change processes [65]. In TVM, the *kosmos* component is important in situating the implementation of the *corpus* and *praxis* within a framework of care and respect for non-human nature. For example, in the Mapuche *kosmos*, the presence of the *ngen* serves to guide proper practices as regards water, its care and use [52]. In view of this, different public policies and government programs related to *campesinos* should include their *kosmos* as a central element [52, 56]. In terms of livestock production, this would permit the joint development of sustainable and bioculturally appropriate strategies. In addition, TVM serves to diversify health management options and it is, therefore, essential to validate the effectiveness of the different plant species. In this context, a social validation approach [66], the use of methods that combine participatory workshops, non-experimental validation [60], and in vitro and in vivo tests to determine therapeutic doses and toxicity levels [67] are alternatives for advancing in the safe and effective use of TVM.

## Conclusions

Our results indicate the presence of diverse knowledge, practices, and beliefs about the traditional veterinary medicine (TVM) that is safeguarded by Mapuche and non-Mapuche *campesinos* in the southern Chilean Andes. Various processes of socio-environmental change associated with agricultural modernization, reductions and changes in land ownership, and climate change are permeating ethnoveterinary knowledge, practices, and beliefs. This explains the persistence and/or loss of use of TVM as well as processes of assimilation of new practices and hybridization, indicating that TVM, far from being static, is constantly adapting. This study offers new perspectives that broaden the range of animal and health management alternatives for revitalizing and increasing awareness of bioculturally diverse livestock farming able, among other things, to navigate the current times of crisis. It is imperative to implement extensionist government programs that are culturally appropriate and respect and consider TVM in its different expressions, together with the promotion of sustainable and nature-friendly livestock *praxis*. In this context, advancing in TVM validation processes would facilitate the safe and effective use of therapeutic plant species.

## Data Availability

To request materials related to this study, contact Fernanda Olivares (fernandaolivaresmd@gmail.com).

## Footnotes

1 Frequency was measured as the number of mentions of the practice by type of production species.
